# Halocline boundary layer restricts the vertical distribution of the box jellyfish *Tripedalia cystophora*

**DOI:** 10.1242/jeb.251708

**Published:** 2026-05-26

**Authors:** Jan-Frederik Freiberg, Niels Röhrdanz, Hermann Kohlstedt, Jan Bielecki

**Affiliations:** ^1^ Electrical Engineering and Information Engineering, Faculty of Engineering, Kiel University, Kiel 24143, Germany; ^2^ Institute of Physiology, Faculty of Medicine, Kiel University, Kiel 24118, Germany

**Keywords:** DeepLabCut, Cnidaria, Stratification, Medusae imaging, Behavioural analysis

## Abstract

Haloclines – sharp salinity gradients frequently formed after heavy rainfalls in coastal habitats – can act as barriers for weakly swimming plankton, but their biomechanical constraints on relatively adept swimmers, such as cubozoan jellyfish, remain unexplored. We examined the vertical distribution of *Tripedalia cystophora* before and after establishing an artificial halocline (35→22 PSU) in an experimental swimming arena. After halocline formation, animals repeatedly entered the gradient layer but did not ascend beyond its upper boundary, despite repeated upward trajectories towards the gradient layer, indicating no obvious avoidance response. A hydrodynamic model supported these observations, demonstrating that stratification drag markedly increases thrust dissipation. Thus, centimetre-scale haloclines impose physical constraints that prevent *T. cystophora* from accessing surface waters through reduced effective upward momentum, rather than through behavioural change. Because the underlying hydrodynamic principles are general, we propose a stratification hypothesis to explain how sharp density gradients shape the vertical distribution of some aquatic animals.

## Teaser

**Highlighted Article:** In the presence of a density gradient, *Tripedalia cystophora* is vertically confined and unable to cross the halocline boundary layer. This is supported by direct observation and physical modelling.

## INTRODUCTION

Vertical stratification of water bodies in marine or coastal environments affects the distribution and mixing of nutrients (Ledwell et al., 1993), the availability of metabolically important dissolved gases (Jeong et al., 2024) and the distribution of zooplankton (Lougee et al., 2002; Lampert, 2005; McManus and Woodson, 2012; Tee et al., 2021), thus shaping predator–prey dynamics of local organisms. The vertical stratification of water can result from density gradients, produced by differences in temperature (thermoclines) or salinity (haloclines). The stability of a halocline is defined by two counteracting forces: a mixing energy input that erodes the density gradient, and a stabilising buoyancy that acts with the density gradient to maintain stratification. The ecological significance of haloclines is based on the diminution of convective motions, leading to critical physical characteristics such as heat-insulating effects, especially prominent in arctic environments (Hull et al., 1988). Previous studies have extensively explored the physical dynamics of haloclines. Svensson (1980) used tracer-diffusion tests to investigate the energy dynamics and stability of haloclines in Norwegian fjords, providing foundational insights into their ecological impact. In coastal areas, large amounts of freshwater runoff or river plumes can lead to the vertical stratification of the water bodies (Ozaki et al., 2021), affecting the physical conditions in these habitats. The foraging efficiency and habitat preference of organisms is affected by stratification. Animals may be unable to traverse haloclines or detect haloclines and display avoidance responses.

There are two widely accepted theories explaining the spatial preferences of potential animals in the presence of a barrier: the preference hypothesis and the buoyancy hypothesis. The preference hypothesis suggests that animals migrate with their prey to areas of higher food abundance or actively evade unfavourable conditions (Pihl et al., 1991). In contrast, the buoyancy hypothesis states that weakly swimming schyphozoan jellyfish are passively restricted to the water layer that matches the buoyancy of their internal osmolality (Suzuki et al., 2018).

Contrary to schyphozoan jellyfish, cubomedusae are active and agile swimmers, capable of swimming against moderate currents (Stewart, 1996; Buskey, 2003; Satterlie, 2016). Their primary mechanism of propulsion is the contraction of the bell and the generation of an adjustable jet stream through the movable velarial aperture (Daniel, 1983; Petie et al., 2011; Colin et al., 2013). The cubomedusa *Tripedalia cystophora* exemplifies this swimming proficiency and exhibits feeding preference behaviour, as it passively hunts copepods in light shafts created by sunlight penetrating the foliage of *Rhizophora mangle* trees (Stewart, 1996; Buskey, 2003). This visually guided behaviour leads the animals to occupy surface waters, where they locate and track the light shafts rather than the prey itself (Garm et al., 2011, 2012; Bielecki and Garm, 2018). Intuitively, *T. cystophora* should adhere to the preference theory of distribution in the presence of a halocline barrier. Yet our own field observations in the Everglades National Park suggest that *T. cystophora* remains below haloclines formed by transient freshwater runoff, which may prevent access to the water surface even under strong sunlight – an otherwise surface-seeking cue – and thereby motivated the experimental tests presented here. This is congruent with findings of other organisms that are confined below vertical stratification barriers, e.g. *Pelagia nocticula* and *Sarsia tubulosa* (Arai, 1973; Greer et al., 2018).

We investigated the vertical distribution of *T. cystophora* in the presence of an introduced halocline to determine whether haloclines physically constrain vertical migration by exceeding the kinetic capacity of *T. cystophora*. By direct observation, we compared the vertical distribution of *T. cystophora* between uniform salinity conditions and in the presence of a halocline. We found that the animals were unable to traverse the halocline and reach the region above the barrier. The calculation of relevant physical parameters supported these findings. In line with our results, we propose a novel hypothesis of animal interactions with pycnoclines: the stratification hypothesis.

## MATERIALS AND METHODS

### Model organism

All animals used in this study were adult medusae of *Tripedalia cystophora* Conant 1897, with bell diameters ranging from 8 to 12 mm. Originating from La Parguera, Puerto Rico, specimens were kept in culture at Kiel University. Juvenile jellyfish were transferred from polyp tanks to 250 litre tanks with a circular flow system, maintained and cultured in North Sea water at 35 PSU at a temperature of 28.0–28.5°C under a 12 h:12 h light:dark cycle. The jellyfish were fed Aqua Biotica orange+ (Mrutzek, Ritterhude, Germany)-enriched *Artemia* sp. and reached adult size within approximately 2 months. A total of 18 medusae were used for the experiments.

### Behavioural protocol

**Fig. 1.**
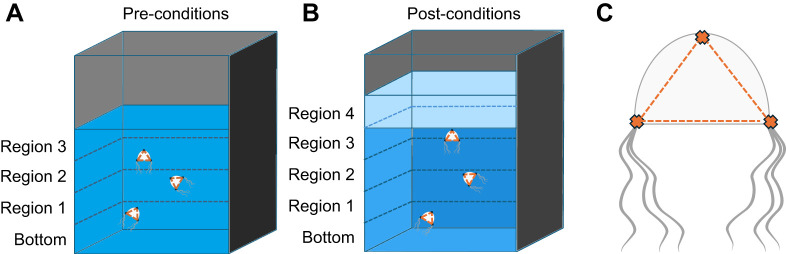
**Experimental arena used to study the influence of a halocline on the foraging behaviour and vertical distribution of *Tripedalia cystophora*.** (A) The arena was filled with 35 PSU North Sea water (28.0°C) to a depth of 20.5 cm and divided into four regions in pre-conditions. (B) During post-conditions, a layer with low-salinity water (22 PSU, 28.0°C) was added on top and termed region 4. The experimental arena was covered in black foil on three sides to eliminate light stimuli from the sides. The animals were allowed to behave freely in the arena and tracked automatically using DeepLabCut software. The algorithm superimposed a virtual skeleton consisting of three markers (bell top, left corner and right corner) onto the medusae (C), and the position of the markers was extracted to determine the position of the animals and compare the vertical distribution between pre- and post-conditions.

During the trials, the animals were transferred to an experimental arena (rectangular tank) measuring 24×24×30 cm (width×length×height), filled with North Sea water at the same temperature as their home tank. The experimental arena was prepared by covering three sides of the arena in black foil to eliminate light stimuli from the sides. The bottom of the arena was lined with black granules to reduce light reflection (Aqua Pacific, Southampton, UK). The tank was placed in a dark room, illuminated solely by an LED light source positioned directly above, creating a circular light shaft. A 75 W heating element (Eheim, Deizisau, Germany) was placed in one corner of the tank to maintain the 28.0–28.5°C water temperature. The experimental arena was filled to a water depth of 20.5 cm (measured above the gravel) with seawater (12.38 litres, 35 PSU, 28.0°C). For each trial, three adult medusae were transferred to the experimental arena and allowed to acclimate for 10 min (Fig. 1A). Following this acclimation period, the box jellyfish were recorded for 10 min under these baseline conditions, referred to as the pre-halocline phase. Subsequently, a halocline was introduced by gently pouring seawater (2.88 liters, 28.0°C) with a salinity of 22 PSU into a spoon to disperse the water and prevent mixing with the underlying water, increasing the water depth to 25.5 cm (Fig. 1B). A salinity of 22 PSU was chosen, reflecting a mean salinity of values observed during field work in the Everglades. The position of the previous water–air interface was defined as the position of the theoretical halocline. The actual position of the halocline was inferred from the behaviour of the animals. After creating the halocline, the medusae were recorded for an additional 10 min, referred to as the post-halocline phase. The arena was monitored using a USB camera (ELP, Shenzhen, China) mounted on a tripod at the vertical position of the halocline (20.5 cm) with a sampling rate of 24 frames s^−1^ and a post-processing resolution of 231 µm pixel^−1^. To account for halocline variability and to analyse the surface-seeking behaviour of the box jellyfish, the frequency of bell contacts with the water–air interface (pre-conditions) and the high salinity–low salinity interface (post-conditions) was tracked, and the exact halocline height was determined for each trial.

### Video and behavioural data analysis

For the analysis, the experimental arena was virtually divided into three equally sized horizontal layers (each 5 cm in height) located below the phase transition (air–water interface) for the pre- and post-halocline phases. The width of the layers equals the width of the low-salinity layer added before the post-halocline phase, which was defined as region 4. The lower region of the experimental arena, below regions 1–3 (or 1–4), was designated the bottom region.

In this study, AI software DeepLabCut v2.3.9 was employed to track jellyfish movements in the experimental arena. DeepLabCut utilises deep learning techniques to automatically produce swim trajectories of the animals during trials. A multi-animal model was trained with the following parameters: architecture: ResNet-50; augmentation: imgaug. The network was initially trained for 115,000 iterations and subsequently refined with an additional 100,000 iterations, using 1400 frames for the initial training and 2880 frames for the refinement phase. The initial frames were extracted from seven video recordings, each containing three animals, using a *k*-means algorithm. These frames were then manually labelled with a three-point skeleton to minimise tracking noise, representing the bell of the jellyfish (see Fig. 1C; bell top, left corner and right corner).

Using custom Python scripts, we quantified the time each animal spent in the designated regions and compared the results between trials with and without the induced halocline (Tables 1 and 2). We defined the number of contacts with the phase transition (CPT) as the total number of contacts between the top of the jellyfish bell and the phase transition zone. In dimly lit regions, the tracking algorithm sometimes failed to detect the animals, yielding NaN (not a number) values in the .csv file output. Our Python script excluded NaNs and markers with a likelihood value below 0.6 (see Fig. S1 for performance).

**Table 1.** Mean time spent in experimental regions by medusae in pre- and post-conditions

	Mean time spent (s)		Standard deviation		
Region	Pre	Post	Pre	Post	*P*-value
Bottom	23.74	71.46	53.25	128.50	0.17
1	146.00	88.51	154.13	109.77	0.22
2	149.70	78.89	87.38	119.67	0.06
3	258.40	298.00	134.36	247.22	0.56

The experimental arena was divided into four regions, and the mean time spent in designated regions was compared between pre- and post-conditions. The medusae spent most of the time in the top region (Region 3). No significant difference in time spent in each region was detected between the two conditions. A *t*-test was used to compare the vertical distribution of the medusae in both conditions. Data for all individuals (3 medusae in 6 recordings) in each video were pooled, and a mean value was subsequently calculated.

**Table 2.** Mean time spent in the top regions in pre- and post-conditions

Mean time spent (s)		Standard deviation		*P*-value
Region 3	Region 4	Region 3	Region 4	
304.36	0.00	175.48	0.00	<0.0001

The time spent in the top regions in both conditions was compared (region 3 for pre- and region 4 for post-conditions). The animals were unable to traverse the density gradient, as no time was spent in region 4 (corresponding to the layer of low salinity water) of the experimental arena in post-conditions. A two-sided *t*-test was conducted to determine the significance of any differences in the duration each animal spent in the topmost regions of both conditions. The data for the individuals in each video were aggregated per video, and the mean value was subsequently calculated.

The position of the lower limit and the width of the halocline varied between the experiments. The position of the halocline was therefore determined separately for every trial and inferred from the behaviour of the animals. In each video, the animals were manually tracked and their interaction with the halocline (or its lower limit) was determined. A histogram of the vertical position of the animals of every trial was created and analysed (see Fig. S2). An event of interest was defined as either (i) a sudden deceleration resulting in an unusually short coasting distance following a contraction, or (ii) horizontal traversal along the barrier. The arithmetic mean value of the positional data acquired by observation of the interactions of the medusae with the stratification barrier was defined as the halocline lower limit (HLL) of the actual halocline, and the maximum positional value as the HLL of the halocline plus the maximum penetration depth of the animals into the halocline. Using a *t*-test, the interactions of the animals with the putative HLL were compared with the collisions of the jellyfish with the water–air interface in pre-conditions to assess changes in the surface-seeking behaviour of the animals.

To evaluate differences in vertical distribution under the two conditions, we applied a *t*-test to aggregated data from all animals. To determine whether *T. cystophora* spent significantly different amounts of time in the upper regions of the tank across the two experimental conditions, we also applied an unpaired *t*-test. Because the *t*-test may be biased when assumptions of homoscedasticity are not met, and to reduce the influence of experimenter-defined boundary choices, we additionally performed a permutation test combined with a Monte Carlo approximation. This allowed us to test for significant differences in the time spent at specific vertical positions distributed in single-pixel bins within the tank. We considered *P*-values below 0.05 as statistically significant (Kim, 2015).

### Calculation of the potential energy of the halocline and kinetic energy of *T. cystophora*

To evaluate whether box jellyfish can generate sufficient kinetic energy to traverse a halocline, several approaches can be utilised. To assess the energy of the barrier, the available potential energy (APE) of the stratified water column can be estimated (Winters et al., 1995; Tseng and Ferziger, 2001).

This approach disregards the influences of drag and considers only buoyancy and the accompanying restoring force (Yick et al., 2009). We included the buoyant force of the model mentioned above in our mathematical model to estimate the penetration depth of *T. cystophora* into the halocline (a comparison of the performance of both models can be found in the Supplementary Materials and Methods). Furthermore, we considered form drag influencing the decrease in the velocity of the animals. Rearranging the drag equation yields (Batchelor, 2000):
(1)

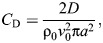
for the coefficient of drag (*C*_D_), where *D* is the drag force, ρ_0_ is the fluid density, *v*_0_ is the relative speed between the animal and the surrounding water (approximated here by the tracked swimming speed), and *a* is the base radius.

The box jellyfish encounter not only a form drag that acts as an opposing force on the movement, but also a stratification-induced force called ‘viscous stratification resistance’, which, in a stratified column, is caused by displacing fluid packages of higher density against gravity and the density gradient into areas of lower density. Instead of calculating an energy balance between the APE of the halocline and the kinetic energy of the jellyfish, we solve a vertical momentum equation:
(2)

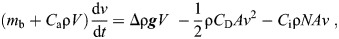
with tissue mass *m*_b_, added-mass coefficient *C*_A_, Δρ as the density difference between the animal and ambient water, ρ as the density of the water, drag coefficient *C*_D_, frontal area *A*, buoyancy frequency *N*, linear baroclinic drag coefficient *C*_i_ and vertical speed inside the halocline *v*(0)=*v*. The viscous stratification resistance *C_i_NAv* is abbreviated as *K*, the buoyant force Δρ***g****V* as *a* and the form drag 1/2ρ*CDAv*² as *D*. Several studies consider the drag coefficient for spheres and hemispheres and, depending on Reynolds number (*Re*), smooth hemispheres have a *C*_D_ in the range of 0.42 (Hoerner, 1965; Daniel, 1983). The coefficient for the linear baroclinic drag (*C*_i_) can be calculated as a product of the Reynolds and Richardson numbers (More and Ardekani, 2023). Following the calculations of More and Ardekani (2023) for Regime R3 (inertial adjective), we estimate *C*_i_=0.8. Based on the experimental estimates reported by Daniel (1985) for medusa bell-like (hemi-ellipsoidal) bodies, which yield added-mass coefficients close to *C*_A_∼1.0 for near-hemispherical geometries, we use *C*_A_=1.0 as a reasonable approximation of the bell of *T. cystophora*.

A closed form of Eqn 2 is not feasible without treating the buoyancy or stratification drag as constant. We consider depth-dependent stratification drag and buoyancy and therefore need to solve the equation numerically using scipy.integrate.solve_ivp. The following equation was used to calculate the penetration depth of the animals numerically:
(3)

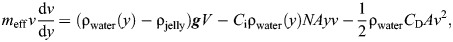
with effective mass *m*_eff_, animal velocity *v*, height-dependent density of ambient water ρ_water_(*y*), animal density ρ_jelly_, gravitational acceleration ***g***, displaced volume *V*, linear baroclinic drag coefficient *C*_i_, buoyancy frequency *N*, frontal area *A*, vertical position *y* and drag coefficient *C*_D_.

We assumed a strictly linear density gradient in the halocline and a single pulse propagation – a single bell contraction of the animal followed by coasting, resulting in a static volume and horizontal expanse of the jellyfish. The box jellyfish approached the halocline perpendicular from below at an initial speed *v*_0_. We treated the box jellyfish as an incompressible hemisphere; the bell did not deform under the influence of drag and buoyancy. We presumed the HLL is homogeneous and does not vary due to internal convection or flows. The model is only valid for *ξ*_max_<Δ*z* (with ξ_max_ being the penetration depth and Δ*z* the halocline thickness); if the animal traverses the halocline, the stratification drag would decrease significantly. Our model assumed a single-pulse movement until the velocity reached zero. Movements with repeated contractions inside the stratification barrier were not observed and are not considered by the model. Furthermore, buoyancy frequency *N*, *C*_i_ and *C*_D_ were treated as constant for our *Re* of approximately 200. We disregarded any additional viscous or turbulent drag effects on the jellyfish.

Calculations were performed for all our induced haloclines using the maximum velocity of the animals. To calculate an upper boundary of the penetration depth, calculations were performed with the highest observed movement speed.

### Mass and initial speed

The mass *M* of the box jellyfish was estimated based on the ambient water density. Most jellyfish are typically considered either neutrally or slightly negatively buoyant, and the exact mass of *T. cystophora* has not been precisely determined. Following the approach used for *Aurelia aurita*, we assumed that the mass of *T. cystophora* is approximately 0.5% greater than the mass of an equivalent volume of water (Yang et al., 2018). Given an almost hemispheric body shape (with radius *r*) of the box jellyfish, the volume of the bell is calculated as:
(4)

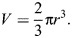
Including water inside the cavity, it moves with the bell and contributes to inertia. The water inside the cavity weighs less than the surrounding tissue; we therefore overestimate the mass of the medusa and its contribution to inertia and penetration depth (*ξ*).

The mass of the box jellyfish was thus approximated using:
(5)

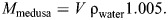
The velocity of the medusa was extracted from the videos used in the tracking experiment. We selected sequences in which individuals moved approximately within the image plane (orthogonal to the camera line of sight) and along an approximately straight path. For each individual, we analysed a 12-frame window (0.5 s at 24 frames s^−1^; Table 3) and calculated the speed as:
(6)

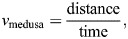
where ν_medusa_ is the velocity of the box jellyfish. The short window was chosen to minimise errors from out-of-plane motion and trajectory curvature inherent to a single-camera setup and to match the single-pulse modelling framework. Depending on contraction rate, a 0.5 s window can span approximately 0.25–1.5 bell contractions (Bielecki, Høeg and Garm, 2013). The maximum observed velocity was chosen for subsequent calculations.

**Table 3.** Movement speed of the medusae

Measurement	*x* coordinate (pixels)	*y* coordinate (pixels)	Distance (mm)	Velocity (m s^−1^)
1	762	416	7.44	0.0149
	743	390		
2	956	685	13.24	0.0265
	940	630		
3	873	569	12.96	0.0259
	906	523		
4	481	614	3.33	0.0066
	493	622		
5	963	620	5.77	0.0115
	964	595		
Maximum velocity (m s^−1^)	0.0265			

To quantify the kinetic energy of the animals and assess whether a traversal of the halocline was possible, the movement speed of the animals was quantified. The maximum velocity of the animals was determined by manually tracking five animals over 0.5 s. The distance travelled by the animals was calculated by determining the length of the vector using *d*=sqrt[(*y*_2_–*y*_1_)^2^+(*x*_2_–*x*_1_)^2^]. The speed was subsequently calculated by dividing the distance by the time: *v*=*d*/0.5 s. The maximum detected velocity was used for the calculations in Eqn 3.

### Use of artificial intelligence

ChatGPT 4o (OpenAI, 2024) assisted in code optimisation and batch processing of video files, enabling the selection and analysis of multiple source files simultaneously and increasing the performance of the algorithms. Furthermore, AI was used to improve the linguistic quality of the text.

## RESULTS

### Behavioural experiment

Following direct observation in their natural habitat, we hypothesised that *T. cystophora* is incapable of breaching the physical barrier posed by a halocline. To test this hypothesis, we monitored *T. cystophora* behaviour in response to an induced halocline and produced vertical distribution trajectories accordingly. To support the observations from the natural habitat, and to estimate whether *T. cystophora* lacks sufficient energy to penetrate a halocline, we calculated the penetration depth of the animals by solving the momentum Eqn 3.

After a 10-min acclimation period in the experimental arena, the box jellyfish displayed natural swimming behaviour. The tracking model demonstrated robust performance (see Fig. S1).

**Fig. 2.**
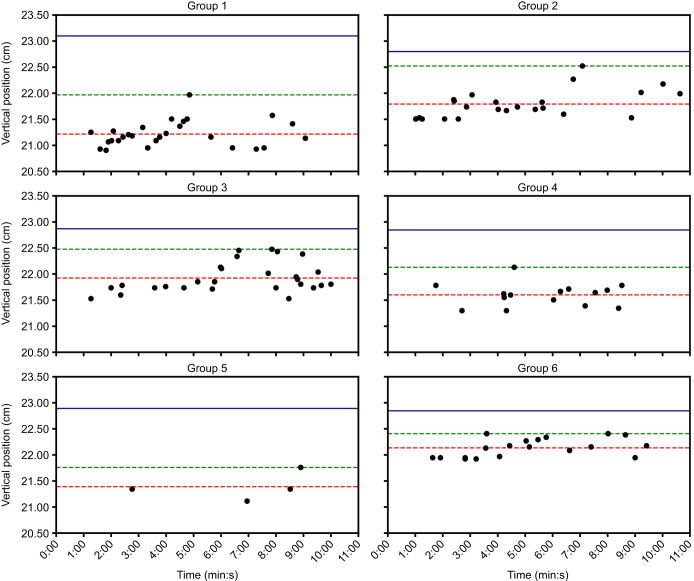
**Quantification and estimation of the vertical extension of the halocline.** Medusae were tracked manually, and their positional data and interactions with the halocline were analysed. The medusae sought the surface and interacted with the halocline, penetrating the density gradient. The mean value of the interactions with the halocline forms the halocline lower barrier (HLL, dashed red line). The maximum penetration depth of the animals is marked by the dashed green line (HLL+penetration depth). The blue line represents the position of the theoretical halocline, identical to the position of the water–air interface in pre-conditions. Six videos, each featuring three animals, were analysed. Dots represent the *x*-values; red dashed line, halocline lower barrier; green dashed line, HLL+penetration depth; blue line, theoretical halocline.

The exact position of the halocline was determined for every trial. The position of the halocline was derived from the behaviour of the animals (Fig. 2). The (*x*,*y*) coordinates of the animals interacting with the putative HLL were extracted and used for subsequent calculations. The width of the halocline in each trial was used to calculate the energy barrier of the stratified water body. The width of the haloclines ranged from 6.93 to 18.71 mm (Fig. 2, Table 4).

**Table 4.** Numerical model used to calculate the penetration depth of the medusae into the halocline

HLL vertical position (cm)	s.d. (HLL) (mm)	Observed halocline width (mm) ±s.d.	Observed penetration depth (mm) ±0.231 mm	Calculated penetration depth (mm)	Approximate deviation (%)
21.22	1.86	18.71±1.86	7.62±0.231	7.16	−6.0
21.76	2.28	10.39±2.28	7.16±0.231	6.46	−9.8
21.89	2.39	9.70±2.39	5.54±0.231	6.37	+15.0
21.65	1.96	12.47±1.96	5.31±0.231	6.68	+25.8
21.39	1.85	11.32±1.85	3.46±0.231	6.56	+89.6
22.13	1.36	6.93±1.36	2.77±0.231	5.94	+114.0

Using Eqn 3, penetration depth was calculated under consideration of form drag and stratification drag. Halocline width was obtained as the difference between the nominal (theoretical) halocline position set during preparation and the experimentally inferred halocline lower limit (HLL; Fig. 2). HLL values are reported as means±s.d. across first-contact events within each trial. The uncertainty of halocline width is therefore ±s.d. (HLL). Observed penetration depth is reported as mean±measurement uncertainty (1 pixel=0.231 mm). Modelled penetration depth is shown as a point estimate. Deviation is computed relative to the observed penetration depth and should be interpreted as approximate.

**Fig. 3.**
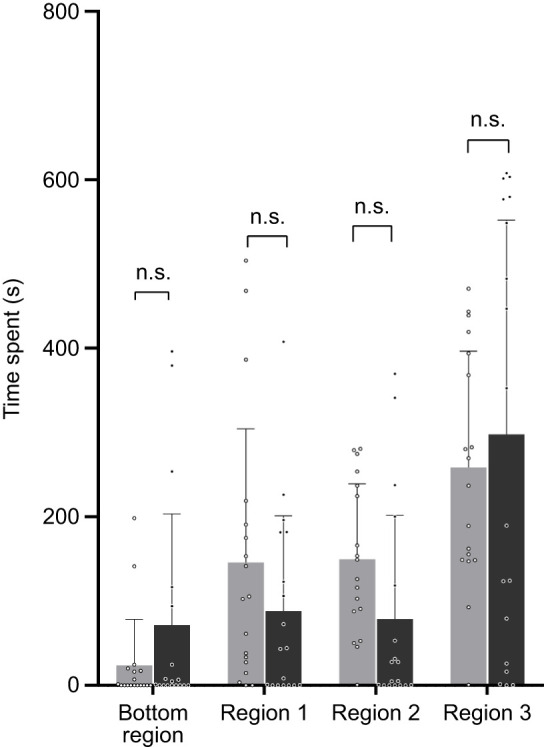
**Behavioural changes of medusae in the presence of a halocline.** The time animals spent in designated regions was analysed and compared. The behaviour of the animals was not affected by the presence of a density gradient. The time spent in the bottom region and regions 1, 2 and 3 did not vary significantly between pre- and post-conditions (*t*-test). The animals were automatically tracked and allowed to behave freely, displaying characteristic surface-seeking behaviour (see time spent in region 3). Three animals were used in each trial, totalling 18 animals across all experiments. Each trial included two conditions: a natural environment (pre-conditions, black) and an environment with an artificially introduced halocline (post-conditions, grey). Animals were recorded for 10 min, and their positions were tracked using DeepLabCut. Positional data were subsequently extracted and analysed in Python. n.s., not significant (*P*>0.05, *t*-test), error bars are ±s.d. Note the consistent distribution despite the halocline.

**Fig. 4.**
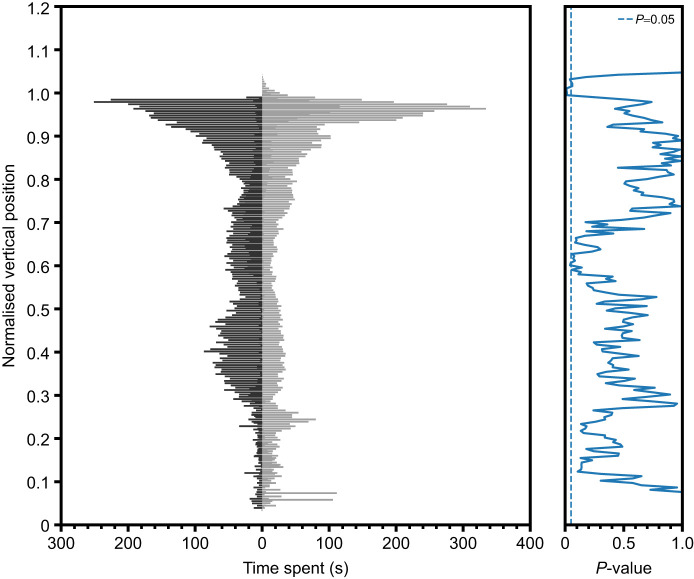
**General behaviour of the medusae in the presence of the halocline.** Animals in both conditions displayed characteristic surface-seeking behaviour, aggregating close beneath the air–water interface (pre-conditions) or the halocline (post-conditions). The vertical distribution of the medusae was statistically indistinguishable between the two conditions. Data were normalised to the theoretical halocline (pre-conditions) or the HLL (post-conditions). Data from all trials and all animals were subsequently pooled for both conditions, plotted as a histogram and a permutation analysis was performed; *P*-values from this analysis are displayed alongside the histogram. A dashed line indicates the significance threshold of 0.05. Frame counts were converted into seconds by dividing by the recording rate of 24 frames s^−1^. Black, facing left: pre-conditions; grey, facing right: post-conditions.

**Fig. 5.**
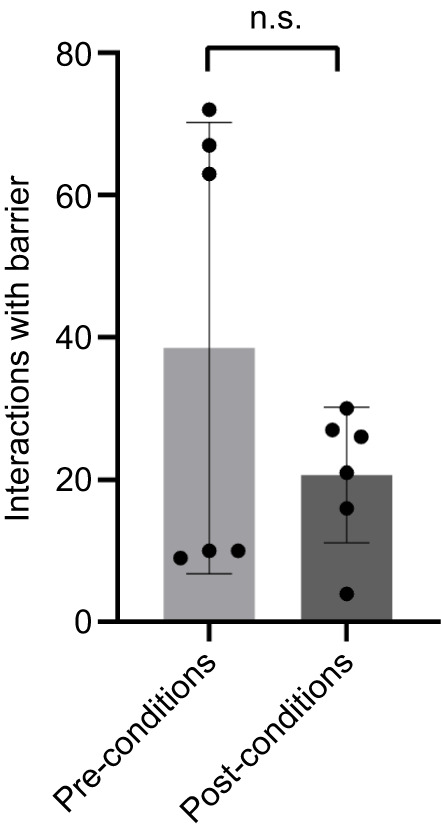
**Exploration effort of *Tripedalia cystophora* in pre- and post-conditions.** The presence of a halocline did not affect the behaviour of the animals, as contacts of the animals with the respective barriers did not differ significantly between the conditions. Animals robustly strived to reach the surface and interact with the barrier. The barrier consists of the water–air interface in pre-conditions or the halocline in post-conditions. Interactions with the barrier in post-conditions were extracted and used to estimate the halocline lower barrier (HLL). A *t*-test was conducted to test for significant differences in the number of interactions with the barrier. n.s., not significant (*P*=0.22). Error bars are ±s.d.

Following halocline introduction, the animals did not significantly alter their vertical distribution; time spent in regions 1–3 was statistically indistinguishable (*t*-test) between the two conditions (Fig. 3, Table 1). To identify potential differences in the vertical distribution of *T. cystophora* below the halocline (pre- versus post-halocline), we performed a permutation analysis. The vertical position-specific permutation analysis revealed no significant changes, except above the halocline boundary, for post-conditions (Fig. 4, right). Normalising the vertical distribution of the animals and comparing the histograms of pre- and post-conditions further revealed no differences in the surface-seeking behaviour of *T. cystophora* in both conditions (Fig. 4, left). We want to emphasise that time spent in the normalised vertical distribution above the HLL baseline in post-conditions does not represent breaches of the halocline or time spent above the theoretical halocline; they are merely the results of penetrations into the halocline and the normalisation of the position of the HLL. Comparable surface-reaching behaviour (to the stratification barrier) was observed under both conditions, as evidenced by non-significant differences in the frequency of CPTs (Fig. 5).

**Fig. 6.**
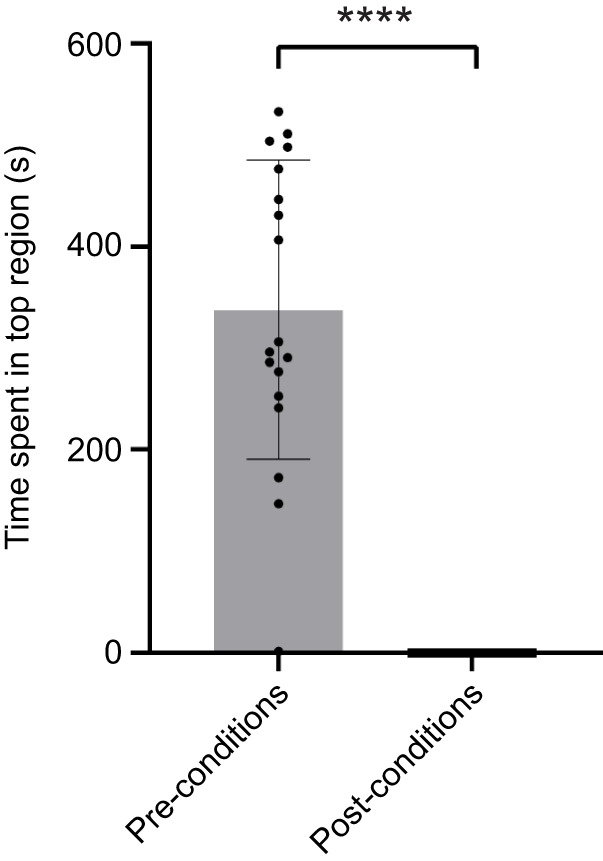
**Time spent in the top region in pre- and post-conditions.** The halocline proved to be an effective barrier for the animals and significantly affected vertical distribution, as medusae were unable to traverse the halocline. Constant observation revealed no time spent in the top region in post-conditions. However, the medusae constantly attempted to reach the surface (see Fig. 4). Three animals were used in each trial, totalling 18 animals across all trials; the arithmetic mean of the time each individual spent in each region was then calculated. The jellyfish were recorded for 10 min, and their positions were tracked using DeepLabCut. Positional data were extracted and analysed in Python (****P<0.0001). Error bars are ±s.d.

We observed a significant difference in the time the animals spent in the topmost regions between the two conditions. Animals did not pass the halocline in post-conditions (unpaired *t*-test: *P*<0.0001, *n*=3, *N*=18; Fig. 6, Table 2); the time spent in region 4 equals 0 s, despite penetration into the stratification barrier and aggregation beneath the interface (Figs 2, 4; Fig. S2).

### Penetration depth into the halocline

In our experimental setup, water with the following densities was used (Millero et al., 1980): (1) density at 35 PSU and 28°C (ρ_1_): 1022 kg m^−3^; and (2) density at 22 PSU and 28°C (ρ_2_): 1017 kg m^−3^.

Vector data for determining the distance travelled by the jellyfish can be found in Table 4. The mass of the medusa was estimated as *m*_b_=269 mg and calculated using the bell radius of 5 mm and the initial speed *v*=0.0265 m s^−1^.

In our case, a simple model estimating the APE of the halocline and thus the kinetic energy-dependent penetration depth of *T. cystophora* overestimates the penetration depth into the stratification barrier (see Table S1).

To mitigate these effects, we added a form drag and a stratification drag term to properly model the penetration depth into the halocline using Eqn 3 and the accompanying parameters: *C*_A_=1.0, *C*_D_=0.42, *C*_i_=0.8, *V*=262 mm^3^, *r*=5 mm, *u*_0_=*v*_0_=0.0265 m s^−1^, ρ_w_=1022 kg m^−3^, Δρ=5 kg m^−3^ and *A*=78.54 mm^2^. Notably, we used literature-based coefficients and selected a conservative (best-case) parameter set that tends to underestimate deceleration, yielding an upper-bound estimate of penetration depth for *T. cystophora*. The penetration depths of the box jellyfish into our introduced haloclines are listed in Table 4.

According to our expanded mathematical model, *T. cystophora*, with an initial velocity of 0.0265 m s^−1^, can penetrate between 5.94 and 7.16 mm into the introduced haloclines. This matches the observed penetration depth, which ranges from 2.77 to 7.62 mm (Fig. 2, Table 4), although it overestimates the penetration depth for sharp haloclines (5.94 mm versus 2.77 mm, +114%). For the most moderate gradient (Δ*z*=18.71 mm), the model predicts a penetration depth of 7.16 mm. This closely matches the observed penetration depth of 7.62 mm (−6%). The precision of our model thereby ranges from −9.8% to +114.0%, encompassing both underestimation and overestimation of the penetration depths of the animals into the halocline. We argue that this is not necessarily an inherent limitation of the model, but rather a consequence of the vastly varying speeds and angles at which the animals approach the halocline. The mathematical model consistently predicted penetration depths smaller than the width of the halocline, thus indicating that *T. cystophora* is unable to pass the entirety of the stratification barrier.

Our behavioural data and the results of the numerical calculation using Eqn 3 indicate that the box jellyfish are physically unable to traverse the halocline with physiologically relevant velocities. When incorporating drag effects on the jellyfish, especially the stratification-specific drag, the predictions of the penetration depth by the mathematical model closely match the penetration depths of animals, underestimating the depth by 6.3% for a halocline of 18.71 mm and overestimating the penetration depth by 114% for a halocline of 6.93 mm. In contrast to the aforementioned buoyancy and preference theory, *T. cystophora* does not actively avoid the low-salinity layer. Changes in body density in response to salinity have been reported to occur on the scale of hours (Mills, 1984), whereas in our experiments the animals encountered low ambient salinity only on short timescales. We therefore propose a novel stratification hypothesis that explains the vertical distribution of animals in the presence of a pycnocline, incorporating form and stratification drag.

## DISCUSSION

In this work, we show that an introduced halocline forms an effective horizontal barrier for the cubozoan *T. cystophora*. All animals were constrained to the water below the halocline throughout all trials. Medusae in both conditions displayed surface-seeking behaviour, clustering immediately below the interface, without a significant difference between the conditions. The halocline posed a physical barrier for the animals, as no time was spent above the theoretical halocline position (*P*<0.0001; Table 2, Figs 2 and 6). Solving a momentum formula that includes quadratic form drag and stratification-enhanced drag (Eqn 3) predicts penetration depths of *T. cystophora* into the halocline, matching the observed values with deviations caused by varying initial speeds and approach angles (Table 4).

Our data indicate that strong, active swimmers, such as *T. cystophora*, become passively confined in the presence of a stratification barrier, if the density gradient exceeds their thrust capabilities (Fig. 6). Previous studies have demonstrated that the driving influence on the aggregation and distribution of plankton in the presence of a stratification barrier is the density gradient, rather than the difference in salinity itself (Harder, 1968). This is consistent with previous studies that have demonstrated the distribution of zooplankton to be significantly affected by the presence of a halocline. Lougee et al. (2002) have demonstrated changes in the migration pattern in the presence of a halocline for several species of zooplankton, some taxa accumulating inside or even above the halocline by modulating buoyancy via gas vacuoles or lipid stores (Alcaraz and Strickler, 1988; Lougee et al., 2002; Campbell and Dower, 2003; Davenport and Healy, 2006). Our findings are supported by observations of the vertical distribution of *A. aurita*, whose weak swimming keeps it trapped below pycnoclines (Churnside et al., 2015; Suzuki et al., 2015)*.* Strong indications suggest that the buoyancy hypothesis best explains the vertical confinement of *A. aurita* in the presence of a pycnocline (Suzuki et al., 2018). In contrast to *A. aurita*, *T. cystophora* is a far more agile swimmer, routinely ascending sunlit shafts in mangrove lagoons (Stewart, 1996; Buskey, 2003). The introduction of the low-salinity layer to the experimental arena and the presence of a stratification barrier did not affect the behaviour of the medusae (see Figs 4 and 5): time spent in regions 1–3 (Fig. 3) and contact frequencies with either the air–water or halocline interfaces were indistinguishable between pre- and post-conditions (Fig. 5). Histograms of the vertical distribution of animals in post-conditions confirm pronounced aggregation a few millimetres below the barrier (see Fig. 4; Fig S2A–F). The inability of the medusae to traverse the halocline is due to physical inability, not behavioural changes. To minimise uncertainty in the estimated HLL, we derived HLL from a kinematic proxy rather than from visual inspection of the interface. Specifically, HLL was inferred from the barrier-contact events of the medusae (Fig. 2), defined as either (i) a sudden deceleration resulting in an unusually short coasting distance following a contraction, or (ii) horizontal traversal along the interface. Here, ‘sudden deceleration’ was defined as a rapid decrease in instantaneous swimming speed over a short time. This approach provides a reproducible estimate of the interface position across individuals and trials. Direct profiling (e.g. with a CTD or conductivity probe) would provide a more direct measurement of halocline thickness and gradient strength; however, we expect that probe insertion could disturb the centimetre-scale stratification in our small tank setup and therefore consider such measurements a valuable extension for future work. The tracking algorithm showed high precision in the regions of interest (Fig. S1), and the virtual skeleton superimposed on the bell by DeepLabCut fluctuated negligibly.

The findings of our behavioural experiment are supported by modelling the box jellyfish biomechanics (Eqn 3). A momentum equation, which regards stratification drag and form drag, consistently predicts penetration depths smaller than the width of the halocline; the animals are thus unable to traverse the halocline (Table 4). Previous works about stratified mixing in both atmospheric and oceanic flows (Winters et al., 1995; Tseng and Ferziger, 2001) show that form drag and stratification drag rapidly dissipate momentum within sharp gradients. We adapted the form-drag coefficient *C*_D_ from Hoerner (1965) and Daniel (1983). Treating the bell as an idealised smooth hemisphere provides a first-order approximation for *C*_D_ in the absence of species-specific drag measurements. The stratification-drag coefficient *C*_i_ was taken from the regime-based estimates of More and Ardekani (2023). Although alternative geometric assumptions (e.g. deviations from a smooth hemispherical shape) would shift the exact coefficient values, our conclusions are robust to plausible parameter variation because the model is evaluated under a deliberately conservative (best-case) parameterisation that tends to underestimate deceleration and thus overestimate penetration depth. Our model can underestimate and overestimate penetration depth depending on the animal's kinematics at halocline entry (most importantly initial upward speed). Importantly, even under best-case assumptions that maximise predicted penetration, the numerical solution consistently predicts penetration depths smaller than the vertical span of the halocline. We therefore conclude that the observed confinement is explained by physical constraints imposed by stratification rather than by behavioural change. Our mathematical model only allows single pulse propagation; a sequence of pulses could possibly enable a deeper penetration into the halocline. It is noteworthy that multi-pulse propagation was not observed in the experimental setup and field observations in the Everglades did not yield animals located above the halocline. The post-impulse model describes halocline interaction as ballistic coasting under buoyancy and drag, including added mass and a stratification-dependent drag, and therefore does not represent cyclic propulsion with repeated pulses. Timing-dependent hydrodynamic mechanisms as resonance and wake capture/passive energy recapture (PER) can contribute to locomotion performance in other taxa and are not resolved by our formula (Gemmell et al., 2013, 2018; Hoover et al., 2019). Although *T. cystophora* is able to modulate contraction frequency in response to visual input (Petie et al., 2011), PER/wake-capture contributions have not been quantified for this species. Mechanistic testing of wake-based strategies at a halocline would require dedicated flow-field measurements in stratified fluids and is therefore beyond the scope of the present study.

Our mathematical model for single pulses provides a more accurate description of the behavioural data than simply incorporating the available potential energy of the stratification system (see Supplementary Materials and Methods) and can be extended to weak-swimming cnidarians to model the vertical distribution in the presence of a sharp density gradient, thereby allowing the modelling of predator–prey dynamics. Previous work has demonstrated that predation risks trigger a diel vertical migration in copepods (Möller et al., 2020). In contrast to the zooplankton studied by Lougee et al. (2002), the vertical distribution of the copepod *Calanus pacificus* was not influenced by the presence of a density gradient, but presumably other chemical cues, such as nutrient density (Cayson, 2018). This can lead to scenarios where a density gradient effectively separates the predator (*T. cystophora*) from its prey, decreasing the food availability for medusae. This can be applied to other animals that are greatly influenced by the presence of haloclines (Graham, Pagès and Hamner, 2001; Malej et al., 2007). In contrast to the buoyancy theory, the density of the box jellyfish is not the main factor keeping them from accessing the water surface (Jacobsen and Norrbin, 2009; Suzuki et al., 2018), but rather the generated momentum and the stable stratification of the water body. Because the medusae did not traverse the halocline, prolonged residence in the 22 PSU surface layer – and thus internal equilibration to the lower ambient salinity – is unlikely. Instead, animals only entered the gradient transiently before returning to the 35 PSU layer, such that their effective density (and the buoyancy term Δρ***g****V*) is expected to remain close to that of 35 PSU-acclimated individuals during these encounters. We nevertheless acknowledge that complete equilibration over hours (Mills, 1984) could reduce the buoyancy penalty and potentially facilitate deeper penetration; however, this would require sustained exposure to the low-salinity layer, which was not observed here. The medusae try to reach the surface and do not actively avoid the interface. We therefore propose a novel hypothesis to explain the vertical distribution of actively swimming *T. cystophora* in the presence of a pycnocline. Medusae are vertically confined when the strength of the stratification barrier exceeds the momentum and kinetic energy of the box jellyfish; the stratification drag predominantly mediates the strength of the barrier. Thus, we refer to this hypothesis as the stratification hypothesis.

*Tripedalia cystophora* remains trapped below a halocline as soon as the density gradient demands more thrust than the jellyfish can generate. Here, tracking freely behaving animals confined by a 35–22 PSU barrier revealed no full crossings. Behavioural consistency in both conditions (Figs 3 and 5) indicates that the primary mechanism of confining the medusae and preventing them from accessing the surface is the stratification barrier, rather than changes in surface-seeking behaviour (Fig. 4). As discussed in previous studies, *T. cystophora* does not visually target individual prey (Buskey, 2003; Garm et al., 2016). Consequently, it is unlikely that the absence of prey above the halocline removes visual stimuli that would otherwise promote halocline traversal. Although it remains unclear whether *T. cystophora* can detect prey-derived chemical cues, haloclines strongly suppress vertical mixing and diapycnal transport, thereby limiting the exchange of dissolved substances across the density interface (Ledwell et al., 1993; Ozaki et al., 2021). Taken together, we consider the prey-free experimental design unlikely to constitute a major limitation of the study.

In this work, we unequivocally demonstrate that *T. cystophora* remains trapped below a halocline as soon as the density gradient demands more thrust than the jellyfish can generate. Tracking freely behaving animals confined by a 35–22 PSU barrier revealed no complete traversals of the halocline. Our mathematical model, which considers form drag, stratification drag and energy dissipation, closely predicts *T. cystophora* halocline interactions. This study provides evidence that, under the tested conditions, haloclines can function as effective barriers for *T. cystophora*, consistent with drag-mediated dissipation of thrust. The proposed mathematical model offers a framework to explore how vertical density gradients may influence the vertical distribution of cubozoan predators and, by extension, potential predator–prey interactions. Finally, we propose a novel hypothesis that explains the vertical distribution of *T. cystophora*, which significantly diverges from established theories, such as the preference or buoyancy theory.

## Supplementary Material

10.1242/jexbio.251708_sup1 Supplementary information
