# Supplementary material for: Halocline boundary layer restricts the vertical distribution of the box jellyfish Tripedalia cystophora
Source: J Exp Biol. 2026 May 26;229(10):jeb251708. doi: 10.1242/jeb.251708 (PMC13286346; doi:10.1242/jeb.251708)
Supplement: Supplementary information [file jexbio-229-251708-s1.pdf]

Supplementary materials and methods

Simple APE model

We compared the performance of our model (Eqn. 3) with a simple model that considers the available potential energy of the stratified system and thus the energy needed to overcome the halocline as a barrier (Winters *et al.*, 1995; Tseng and Ferziger, 2001).

Following the calculations for the available potential energy and assuming a linear density gradient, we calculated the APE (equivalent to the work) for small deflections (ξ) by

$$W(\xi) = \frac{1}{2} g \frac{\Delta\rho}{\Delta z} \xi^2 V \qquad \text{(S1)}$$

Solving for the excursion ξ, which equals the maximum penetration depth of the animals, gives

$$\xi(W) = \sqrt{\frac{2\Delta z W}{\Delta\rho g V}} \qquad \text{(S2)}$$

This approach disregards additional energy sinks, such as drag and stratification forces. A comparison of the calculated penetration depths using this approach and Eqn. 1.03 can be found in Table S1.

Journal of Experimental Biology • Supplementary information

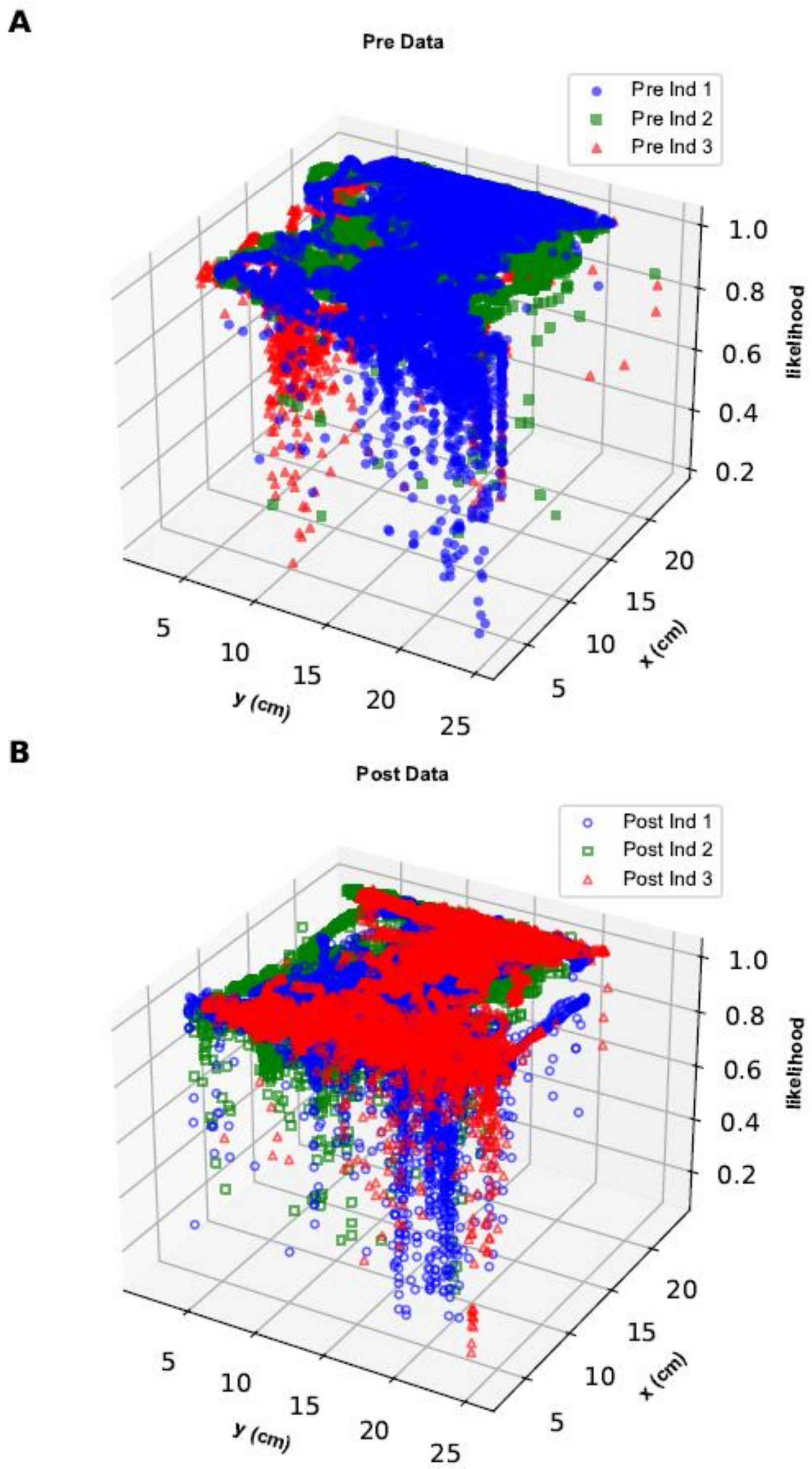

Fig. S1. The tracking algorithm displays high precision in the regions of interest. Most of the superimposed markers of the virtual skeleton display a high likelihood. The likelihood of the markers decreases in the lower regions of the experimental arena (note that the x-axis represents the height of the experimental arena). Translucent medusae and reflections near the substrate impair the performance of the algorithm. All data points from each individual's pre- and post-halocline recordings were combined and down-sampled by a factor of 10 for improved readability. The x-axis (x) represents the width of the experimental arena, while the y-axis (y) corresponds to its height.

Journal of Experimental Biology • Supplementary information

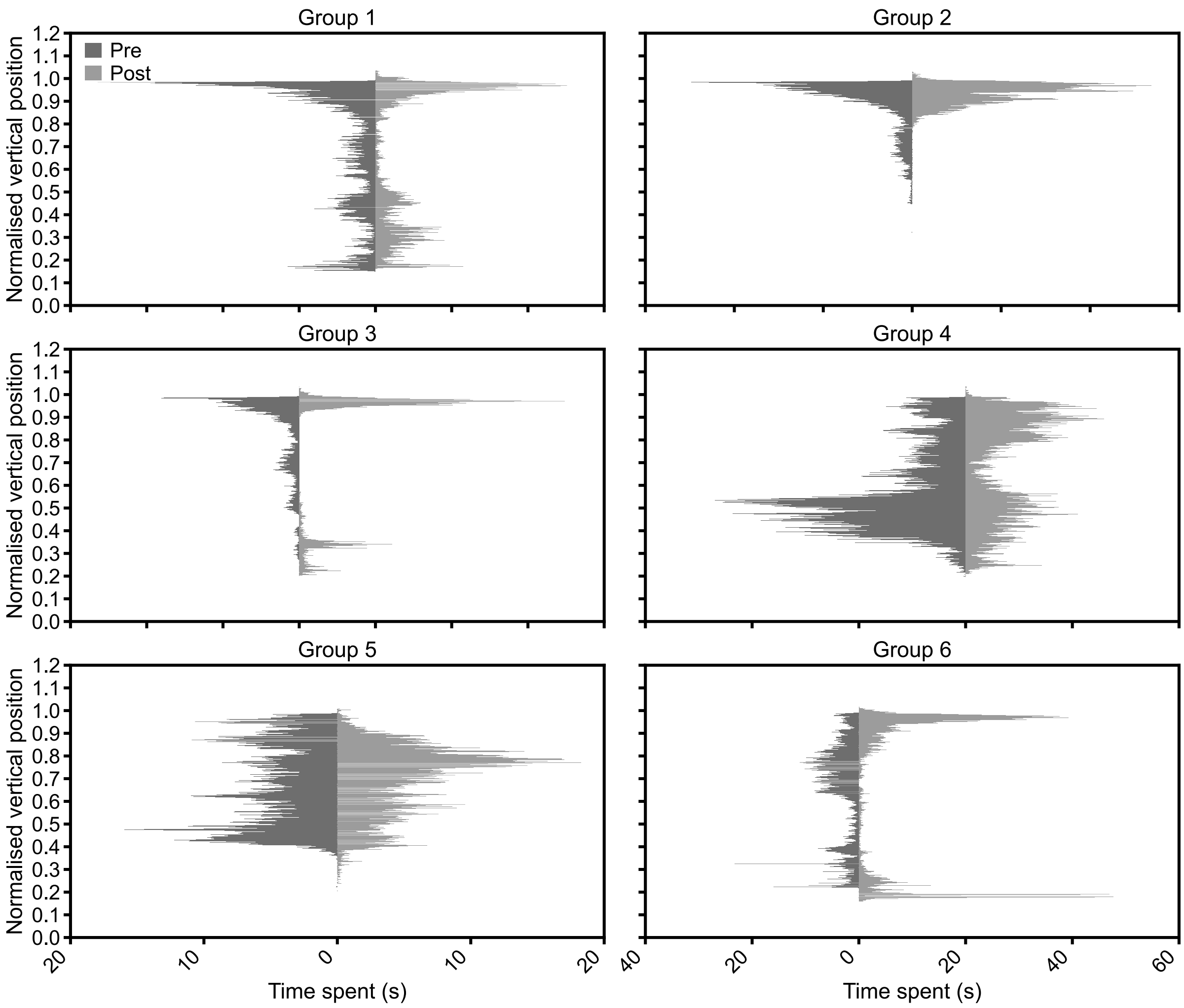

Fig. S2. The medusae display consistent surface-seeking behaviour in both conditions. The animals aggregate near the barrier (water-air interface or halocline). The vertical distribution of animals below the respective interfaces remains constant; histograms for both conditions show comparable distributions. The positional data has been normalised using the water-air interface (pre-conditions) or the estimated HLL (post-conditions).

Journal of Experimental Biology • Supplementary information

Table S1. The mathematical model incorporating form drag and stratification drag is superior to the simple APE model. The calculated penetration depth of Eqn. 3 is far more accurate than the calculated penetration depths of the analytical APE model. Both models considered the same velocities, mass and volume of the medusae. The APE model predicts complete traversals of the halocline, which was not observed during the behavioural experiments. The numerical approach (Eqn. 3) consistently predicted penetration depths smaller than the vertical spread of the halocline.

Observed Halocline width [mm]	Observed Penetration Depth [mm]	Calc. pen. depth (Analytical APE) [mm]	Calc. pen. depth (Numerical, max v) [mm]
18.71	7.62	14.52	7.16
10.39	7.16	10.82	6.46
9.70	5.54	10.45	6.37
12.47	5.31	11.85	6.68
11.32	3.46	11.29	6.56
6.93	2.77	8.84	5.94

Journal of Experimental Biology • Supplementary information
